# A longitudinal qualitative study examining the factors impacting on the ability of persons with T1DM to assimilate the Dose Adjustment for Normal Eating (DAFNE) principles into daily living and how these factors change over time

**DOI:** 10.1186/1471-2458-11-672

**Published:** 2011-08-30

**Authors:** Dympna Casey, Kathy Murphy, Julia Lawton, Florence Findlay White, Sean Dineen

**Affiliations:** 1 School of Nursing & Midwifery, National University of Ireland, Galway, Ireland; 2 Public Health Sciences section, Centre for Population Health Sciences, The University of Edinburgh, Medical School, Teviot Place, Edinburgh, EH8 9AG, Scotland; 3 Diabetes UK Northern Ireland, Bridgewood House, Newforge Lane Belfast BT9 5NW Northern Ireland; 4 Endocrinology and Diabetes Day Centre, University Hospital Galway and Department of Medicine, National University of Ireland, Galway, Ireland

**Keywords:** Type 1 diabetes, structured education programmes, DAFNE, chronic disease self management, longitudinal qualitative research

## Abstract

**Background:**

The literature reveals that structured education programmes, such as DAFNE, result in many positive outcomes for people with Type 1 diabetes including a decrease in HbA_1c _levels and reductions in hypoglycaemia. While there is evidence that some of these outcomes are maintained we do not know at present what factors are most important over time. The study aim was to identify the key factors impacting on persons with Type 1 diabetes ability to assimilate the Dose Adjustment For Normal Eating (DAFNE) DAFNE principles into their daily lives and how these factors change over time.

**Methods:**

This is a longitudinal descriptive qualitative study. Interviews were undertaken with 40 participants who had attended DAFNE in one of 5 study sites across the Island of Ireland, at 6 weeks, 6 and 12 months after completion of the programme. The interviews lasted from 30 to 60 minutes and were transcribed verbatim. Data were analysed in three ways, a within time analysis, a cross sectional analysis for each participant and a thematic analysis which focused on examining changes over time

**Results:**

Four themes that influenced participants' ability to assimilate DAFNE into their daily lives over time were identified. These were: embedded knowledge, continued responsive support, enduring motivation and being empowered. Support at the 6 month period was found to be crucial to continued motivation.

**Conclusions:**

Understanding the factors that influence people's ability to assimilate DAFNE principles over time into their daily lives can help health professionals give focused responsive support that helps people with diabetes become more empowered. Understanding that continued support matters, particularly around 6 months, is important as health professionals can influence good management by providing appropriate support and enhancing motivation.

**Trial registration:**

ISRCTN79759174

## Introduction

In the island of Ireland there is an estimated prevalence of diabetes of between 5-5.8% [1]. In the context of the Republic of Ireland the Diabetes Federation of Ireland estimates that approximately 12,000 people have Type 1 Diabetes Mellitus (T1DM). Self management skills are essential if persons with type 1 diabetes are to manage their condition effectively [2,3]. One way of developing these skills is through participation in a structured education programme (SEP) [4]. Currently in Ireland the Dose Adjustment for Normal Eating (DAFNE) SEP is being delivered to clients with T1DM, as part of a large 5 year multi-centred randomised controlled trial [5] examining the implementation and evaluation of the SEP and to develop a new model of ongoing care. The qualitative component of the study, which is reported in this paper, focuses on illuminating the factors that influence persons with T1DM self management and in particular examines the factors that influence their ability to assimilate DAFNE principles into their daily lives over time.

## Background

Structured education programmes are defined as planned programmes which are:

"Comprehensive in scope, flexible in content, responsive to an individual's clinical and psychological needs and adaptable to his or her educational and cultural background" [4].

Nowadays there are several SEP's for persons with diabetes delivered across the globe all aiming to enhance self-management skills and enable people to understand how to live with their chronic illness, and work collaboratively with health care professionals. Examples of SEP's include the Diabetes Education and Self-Management for Ongoing and Newly Diagnosed (DESMOND) (Type 2) and the Dose Adjustment for Normal Eating (DAFNE) (Type 1). DAFNE is a SEP for people with Type 1 diabetes delivered over 5 consecutive days by a multi-disciplinary team. The curriculum covers all aspects of living with diabetes but places a strong emphasis on blood glucose testing, carbohydrate counting and matching quick-acting insulin to this. If a person is to assimilate DAFNE principles into the management of their diabetes these key DAFNE components need to be used to guide decision making. The ethos of the programme is empowerment and the aim is to promote self management skills. Approximately 6 weeks following the course DAFNE participants meet to review progress and goals. Delivering education through a structured education format is thought to result in a paradigm shift from the traditional medical model of care to a more empowering model that fosters self-management [2]. Furthermore the evidence is that this is an appropriate education programme and is cost effective [6].

A number of studies have examined the impact of SEPs over time and findings reveal that these educational programmes are associated with improvements in: blood glucose control [7-15]; diabetes management skills and compliance [7,9,12,15] perceptions of self efficacy [16,8]; relationships with assigned doctors [8]; well being and quality of life [11,17] knowledge levels [9,11,12,18] and empowerment [16]. However the main focus of many of these studies has been on clinical outcomes, in particular HBa1c level, rather than self management and the initial improvements in results appears to diminish after 6 months [19]. Few studies have identified what fosters long term sustained improvements in self management or what factors influence self management over time.

Nevertheless, studies have identified key factors that influence diabetic self management and contribute to improved outcomes, albeit not focusing on changes over time. These factors include family support [20-22]; peer support [23-25] and the support of health professionals [26,22]. In particular support from health professionals and good health care provider relationships have been identified as key to successful diabetes management [27-32]. The National Standards for Diabetes Self Management Education (DSME) has also highlighted the need for ongoing sustained support to maintain advances made by patients during the initial DSME programme [2]. They outline a number of strategies to promote ongoing support including reminders about follow up care, on-going education, behavioural goal setting and psychosocial support or connection to community resources.

However, few studies have explored how support works from the patient's perspective and what aspects patients consider useful. Edwall et al. [33] interviewed persons (n = 20) with T2DM regarding the meaning they attached to the consultation with the diabetes nurse specialist (DNS) at their annual check-up. They concluded that patients rely on the DNS for support, in particular in times of crises. Fox & Chesla [34] focusing on chronic illness, examined women's health care provider relationships and how these relationship influenced their health. Eighteen of the twenty five women interviewed had diabetes. They also found that healthcare provider relationships characterised by genuiness, honesty and reciprocal respect and trust resulted in women feeling more secure, empowered, confident and motivated to self manage their diabetes. Oftedal et al. [35] examined T2DM patients perception of the characteristics of support following SEP and how these impacted on patients motivation to self manage. Likewise, they found that the important elements of support provided by health care providers were being emphatic, providing practical advice and information, working in partnership and feeling involved in decision making processes, being provided with accurate and individualised information; and, having continual ongoing support. Similarly Penn [36] et al. found that professional support as well as social support was key to helping patients maintain behaviour change for at least 2 years following a lifestyle intervention. This qualitative study was part of a larger RCT focusing on the impact of lifestyle interventions in preventing the onset of T2DM. Lawton & Rankin [37] also found that the group education process and the interactions between fellow group participants and course educators were important factors influencing changes in self management during DAFNE courses observed in the UK. This study involved interviewing patients (n = 30) as well as observing the delivery of DAFNE SEP courses, to explore the processes and dynamics in relation to how SEPs are delivered and experienced. However the ways in which persons with diabetes may perceive that their support needs change and alter over time, why this might occur and how this might impact on motivation to self manage remains a relatively unexplored area.

Knowledge has also been identified as a pre requisite for positive adjustment to chronic illness and better self management and control [38-40,16,25]. It is generally accepted, however, that after education has been received there is a loss of knowledge over time unless the person uses the newly acquired knowledge in a relevant context frequently [41]. Combining received knowledge with one's own knowledge and experiences of living with diabetes, obtained from a trial and error approach, yields greater understanding, as the person learns how diabetes, insulin and one's body interact [42]. Therefore the extent to which a person is able to reorganise the knowledge and enhance its contextualisation may be predictive of behaviour change, as people try to integrate and embed the knowledge acquired on an educational programme into everyday life [43]. However how a patient's knowledge following a SEP alters and is maintained over time is less clear.

Successful self management also requires motivation, [3,44] and patient empowerment [21,45]. Patient empowerment underpinned by self determination theory stipulates that individuals are naturally motivated to improve their own wellbeing. They are motivated autonomously when they feel they have a choice, free will and there is similarity between the behaviour and their own personal values. Self determination however requires patience all day, every day [42]. A high level of autonomous motivation for self care has been linked with more acceptable HbA1c levels [46] maintenance of a healthy diet and testing blood glucose levels [47]. However few studies have explored patients' perceptions of motivation and the issues that impact on motivation over time.

Personal characteristics and the type of person one is can also influence a person's ability to master self management of diabetes [42]. In particular the literature reveals that people's perceptions of personal responsibility in managing their diabetes is a factor influencing self management [48-50]. Rayman and Ellison [48] suggest a self management continuum whereby a person moves from having knowledge but does not self manage ('pre engaged'), to 'engaging in self management but still struggling to self manage and finally to effective self management ('engaged and adjusting').

Finally, life events also govern whether or not people with diabetes perform or adhere to recommended self management principles [51,49]. Penn [36] et al. found that pre existing co morbidities and social obligations, in particular, are barriers to maintaining changes over time. Other studies reveal that the desire to 'fit in' and 'be normal' make it difficult for young adults with T1DM to routinize self management practices [52,53]. Furthermore, key life transition periods; for example, moving to a new and unfamiliar environment such as university hinders self management [53]. For many people with TIDM dislike of undertaking blood glucose self monitoring [42] injecting in public [46] and fear of hypos [42] are also key barriers to effective ongoing self management. Self management therefore must be understood in the context of life events which vie with the time and concentration that self management requires [54].

In summary, the evidence from quantitative studies suggests that, over time, SEP has the potential to impact positively on patient outcomes. However, these findings do not appear to be consistent, in particular in relation to HBA1c. However, these studies have predominantly focused on quantitative clinical outcomes tending to ignore participants' own accounts and experiences of the self management process. Studies that have explored participants' accounts reveal a number of factors important to the self management process however the ways in which these factors may alter and change over time have not been studied thoroughly yet.

## Methods

Longitudinal qualitative research is most appropriate when the aim is to generate rich data and a deeper understanding regarding people's perspectives and experiences and how and why these may change over time [55]. This approach was therefore in keeping with the aim of this study which was to identify key factors that impacted on patients' ability to assimilate DAFNE principles into their daily lives and how, and why, these may change over time.

### Sample

This qualitative study forms one research arm of the Irish DAFNE Study that commenced in 2006. This larger study is a cluster RCT evaluating the impact of two different methods of follow-up of DAFNE graduates on biomedical and psychosocial outcomes [5].

Five DAFNE centers across the island of Ireland agreed to participate in the qualitative research. A list of all participants from these sites were obtained (n = 192). Utilizing maximum variation to ensure a diversity of perspectives, a purposive sample of 40 participants, approximately 10% of the sampling frame were interviewed at three different time periods, 6-8 weeks, 6 months and 12 months post attendance at DAFNE. Two participants were lost to follow up at the 12 month interview due to illness, therefore a total of 118 interviews were undertaken. Participant age, length of time since diagnosis, and gender are provided in Table 1.

**Table 1:** Participants' age, gender and length of time since diagnosis

	*n (N = 40)*	*%*
**Age**		
**20-30 years**	11	27.5
**31-40 years**	11	27.5
**41-50 years**	13	32.5
**51-60 years**	4	10
**61-70**	1	2.5
**71+**	0	0
		
**Gender**		
**Men**	15	37.5
**Women**	25	62.5
		
**Length of Time Since Diagnosis**		
**2 - < 4 years**	5	12.5
**4 - <6 years**	3	7.5
**7 - < 10 years**	6	15
**10 -20**	11	27.5
**21-30**	8	20
**31+**	7	17.5

### Data Collection

Semi-structured interviews using an interview guide was used to collect the data by two researchers over a 36 month period from 2006-2009. First round interviews focused on participants' initial experiences of participating in the DAFNE programme and initial use of the DAFNE principles. Second and third round interviews focused more on ascertaining how participants were managing their diabetes and exploring their use of the DAFNE principles over time, in real life. These interviews typically began with the question "so how have you been managing using DAFNE principles since we last talked?" These later interviews included questions which explored and verified emerging themes. First round interviews lasted between 45 and 60 minutes, with second and third round interviews being more focused and averaging 20 minutes. All interviews were tape recorded with participants' consent and transcribed in full.

Ethical approval for the study was obtained from the National University of Ireland, Galway Research Ethics Committee (06/MAY/04), Galway University Hospitals (CA-19) and relevant local hospital Research Ethics Committees. Written consent was obtained from all participants and confidentiality was ensured by the removal of all identifying material.

### Data Analysis

There is little consensus as to how longitudinal qualitative research analysis should be undertaken [56]. In this study longitudinal analysis was undertaken guided by Holland et al. [57,57] and three stages of analysis were completed. The first stage was a within time analysis, this entailed analysing each data set at a specific time period, i.e. 6 weeks, 6 months, 12 months. All data were open coded using thematic analysis and then axial coding was used to identify categories [58]. Stage two was a cross sectional analysis which focused on looking for change between points in time for individual cases. The third stage was a thematic analysis that focused on changes over time. The work of Saldana [59] and Lewis [56] were also used to further aid data interrogation (Table 2**and **Table 3). N-VIVO version 8, a qualitative software indexing package, was used to facilitate data management, coding and retrieval.

**Table 2:** Framework for data interrogation in qualitative longitudinal analysis [56]

Key area	Focused questions
Description	What is the type, extent and timing of any change

Location	Who changed their minds and in what contexts

Drivers for change	What were the factors that influenced the changes

Evaluation	What influenced the experience of self management and change or lack of change

Consequences	What was the affect of further changes new directions, loss of opportunity

What do people expect to happen next	What changes to their daily life does it make and what are the consequences

Personal meaning	What was the perceived importance of the change the impact on identity; do they see themselves or their futures differently

Policy meaning-	What makes DAFNE work, what contextual changes are important

Reflections on the researchers influence	

**Table 3:** Guide to qualitative longitudinal analysis [59]

Framing questions
• What is different from one round of data to the next

• When do changes occur through time

• What contextual and intervening conditions appear to influence and affect participant changes through time

• What are the dynamics of participant changes through time

• What preliminary assertions about participant changes can be made as data analysis progresses?



**Descriptive questions**

• What increases or emerges through time

• What is cumulative through time

• What kinds of surges or epiphanies occur through time

• What decreases or ceases through time

• What remains constant or consistent through time

• What is idiosyncratic through time

• What is missing through time



**Analytic and interpretive questions**

• Which changes interrelate through time

• Which changes through time oppose or harmonize with natural human development or constructed social processes

• What are participant or conceptual rhythms e.g. cycles through time

• What is the through-line of the study?

### Rigor

Four criteria were used to ensure rigor: credibility, auditability, confirmability and applicability. Participants' perspectives were reported as accurately as possible, and they were sent a copy of their transcripts and asked to confirm that the content was accurate. Two participants requested a minor change to their transcript following this process. Comments were also invited from medical and nursing experts in the field who confirmed that the findings were consistent with their experiences. A sample of transcripts were analysed independently by two researchers and codes, categories and themes compared and agreed.

## Results

The aim of the research was to identify key factors that impacted on a person's ability to assimilate DAFNE principles into their daily lives and how this changed over time. Four themes were identified being empowered, embedded knowledge, enduring motivation, and continued responsive support. Each theme is described below and, where appropriate, their subcategories.

### Being Empowered

Being empowered described the overall outcome that could be expected when a person committed over time to the DAFNE principles to self manage their diabetes. Being empowered allowed people with diabetes to have control over their diabetes and to take decisions about how they wanted to do this.

*"It's great having that bit of knowledge so that it empowers you to make the decision, now whether you make that decision or not, is not the fault of the DAFNE course, it's the responsibility of the person (P93-039 6 weeks)*.

"...do DAFNE because it does - it empowers you greatly (P51-053 12 months)

Some participants, however, never attained a sense of being empowered as they found self management difficult and struggled with day to day management and control. It was evident for one of these participants that other life events had an impact:

Its, you know, again I mean in theory, everything works, in practice it's a different ballgame...I've been kicked around quite a lot over {name of wife} being diagnosed with cancer..., this DAFNE woman, I mean she seems very good, and I think it works in general terms, it probably would...but I don't think my problem, my stability can be addressed from a DAFNE point of view...(P75 -079-6 weeks)

### Embedded Knowledge

This theme describes how knowledge developed over time through the experience of using DAFNE. Two sub-categories were identified: knowing more and choice and consequence.

#### Knowing more

During the first round interviews all participants agreed that DAFNE had increased their knowledge and had impacted hugely on their diabetes management as it enabled them to make informed choices about food and adjustments to their background or quick-acting insulin and to manage hypoglycaemic events effectively:

"Your doctor would say a long time ago, take a Mars bar, take a mouthful of lucozade, at least I know now, not to take a full Mars bar, I know what a Mars bar is going to do, and that was through the course...before you actually take too much and have a hypo afterwards....I really know a lot more now" (P31-0046 6 weeks)

Knowledge however took time to fully develop as many participants found carbohydrate counting and calculating short-acting insulin doses challenging and reported that it was time consuming to review carbohydrate portions and integrate new foods into their diet. Participants' described the struggle to calculate carbohydrate values at first:

"I can calculate things like potatoes and things like that, but I mean it is a bit...it's a challenge to pick up a packet of something and get your reading glasses on... every packet you pick up is different, but I think probably the biggest challenge is when you're out socially, and you're just wondering what sort of a portion is going to come. And I found a few times that I've kind of miscalculated and then I've got to just take a few more units of quick-acting insulin" (P3-85 6 months)

Over time, participants reported that the knowledge of carbohydrates portions and insulin requirements gradually became more embedded. While it was still challenging at six months to calculate foods by 12 months this was reported as somewhat easier and the time required lessened. This made day to day management easier for most people as they knew the carbohydrate values of most of the food they ate:

*"Yes, I can gauge things better, and I can look at something and know that with the help of the book how many carbohydrates and CPs are in that, and how many {units of}insulin's will I need to take to work the ratios" (P65-069 6 months)*.

You get to judge the portion sizes except when you eat out, because they always give you bigger than what you'd give yourself. So in that respect, like, that's one thing that I do think has made a huge difference. It's just so much faster now because I know what I'm eating. I know what to take. I don't have to spend too much time (P99-014 12 months)

#### Choice and Consequence

Over time, participants suggested that knowledge gave choice and freedom to eat what you liked but they stated that choice was related to consequence. Many participants felt free to eat what they wanted to, but often decided it was not good to do so, either because of the need to take insulin or the potential impact on overall health. They realised that there were consequences to always eating what you liked over time in terms of potential weight gain and more frequent insulin injections.

*But, I know that if I fancy a bar of chocolate I can...I was at a dinner last night in my in-laws. There was a dinner for the family, and when it was over there was dessert, and I could .....the night before I didn't have dessert I didn't fancy it. Last night I did...and I went and I took an injection for it, and I was happy (P114-064 6 months)*.

... but if there was something like, we'll say a piece of cheesecake, because that's something I like, and the middle of the afternoon, to have a piece of cheesecake, well I look at it now and I think ...oh, cheesecake, I'd quite like it but, yes, I'd have to take an injection, and I can't be bothered doing that, so I don't really want it. So it's just...it's given...it seems to have given me a sense of responsibility for eating the food,(P20-62 6 weeks)

if I'm in Tesco and somewhere, and buying say a chocolate, chocolate biscuits or something for the family well I'm looking at the carbs, and like say it's 3 carbs - say it's 30 carbohydrates whatever the chocolate biscuit is - well I wouldn't eat that, because I reckon if I have to take 3 shots of insulin for one little biscuit well I'm not going to eat it. It must be fattening. So I don't bother (P1-.82. 12 months)

Some participants however found that 'being a diabetic' was dominating their life and that life had become less free as a result of DAFNE because they had to think about each bit of food that they ate.

"OK, constant monitoring, and watching, and counting has taken over. My life is not as free as it used to be in so far as you took 4 injections a day, and you just went about your business. But now every morsel of food over 10 grams of carbohydrate requires an injection" (P91-35 6 months)

### Enduring Motivation

Enduring motivation describes the issues that impacted on motivation over time. It divides into three subcategories, trying to get it right, working with uncertainty and preventing complications.

#### Trying to get it right

Initially, participants were enthused and highly motivated to make DAFNE work for them. At this point they believed it was just a matter of sticking with it and as they became more experienced control would be better. By six months, however, some participants were still struggling to make DAFNE work for them. Finding that it does not go to plan resulted in some participants feeling tired, frustrated and de-motivated. What was most difficult for these participants is that they found variability in blood glucose levels despite eating the same foods and injecting the same insulin dose. It was the lack of explanation for why this occurred that was particularly difficult because all the effort seemed to no avail. Over time motivation remained an issue for many participants, some were struggling because they did not understand why things that worked perfectly one week resulted in large fluctuations in blood glucose the next time, therefore, over time, the experiences of participants impacted greatly on their motivation:

*"...every day's different! One day I think everything's going great, the next day it doesn't go as great. Even though I've done the same things - this is where I get annoyed. I think to myself 'why is this like this?' and 'why?'(P16-075 6 months)*.

For other participants, it was evident that life course events also influenced their motivation. These participants described the difficulties of staying motivated and focusing on self and self management issues when faced with serious illness in the family, or other social demands.

I would intend to test now and then start to see how it's going, and then have my coffee. But then I'm going to XXXX for 11 o'clock, and I've got my XXXX to see...and the phone has never stopped ringing, and I have had customers phoning me, and life and my sister's ill. It's life...in the middle of, you know, really I find it really difficult, and I'm self employed and at home on the farm. I should be able to make it work... But by the time that 6 months had gone by I had completely lost - things had happened, you know. My mother went to hospital... so I'm under all this stress... (P06-062 12 months)

*My wife had a baby so...everything was harder to control through that period, and I had - ah - the blood sugar test HbA1c test after that, you know coming into probably 2, 3 months after that, and I would put it down to that really...what I'm saying is if my wife wasn't having the baby it would have been a lot easier do you know...My mind was in another place*.

(P39-0046 12 months)

#### Working with uncertainty

Sticking with DAFNE in times of uncertainty therefore was not easy; however some participants managed to do this and enjoyed the challenge, they were the problem solvers who wanted to work at it to get it right:

*"not everything is going to go by the book, and you have to realise that, and some meals, one time, seem to trigger a high, and if it was something I enjoyed, I would eat it again, and I would suddenly discover it was exactly as you had expected it to be, so there is obviously some other factors, whether your frame of mind, or stress, or whatever is coming into play, but you just have to take that as part of it, its not going to be perfect, ... you're getting stable results, which is encouraging" (P20-81 6 weeks)*

DAFNE therefore required people to be persistent and, at times, to work with uncertainty. The six month interviews were the point at which struggling participants were likely to express most frustration.

...I couldn't keep them balanced {blood sugar levels}. You know they were...they were...(sounds frustrated)... I was going high, I was going high when...and I was convinced I was doing things right, you know I was looking after...I was weighing my...I was doing my food, I was reading the CPs on the back of it. In my own head I was doing everything right (p31-642 6 months)

No matter how much I follow all the...points of DAFNE it still didn't help, but it was just that I was at a loss, you know, my blood sugars were still very high readings, you know, and there was no answer for the high readings, you know. It wasn't like I hadn't taken enough insulin, or I was eating anything more sugary, you know, or the portions weren't being counted right, and I suppose that's why I felt at a loss. So I suppose I found it frustrating (P41-048 6 months)

At twelve months, these participants were more likely to accept that some variability was inevitable, and therefore the impact of uncertainty was less evident on motivation. At 12 months it was evident that participants found that carbohydrate counting and insulin management had become easier through experience and practice.

I was getting' more confident with the DAFNE, I knew how to adjust the things, and I tend to remember the results from the day before or two days before and if I felt they were high you know I would have know how to readjust the Lantus you know. So basically maybe I am becoming after the year just more confident with DAFNE in general you know (P42-048 12 months)

#### Preventing complications

For most participants preventing complications and seeing improvements in their HbA_1c _was an important motivational factor. All participants described the fear of developing complications and the need for good control to prevent this:

I think the huge motivation is to prevent complications, both short-term and long-term, and I know that this works, so you know I will keep going and I will look for other alternatives if I need them (P8-96 6 months)

Over time this remained an important motivational factor and participants at the twelve month interviews still maintained that this was one of the key motivations to remain with DAFNE. Some participants reported that they had experienced fewer hypoglycaemic episodes since commencing DAFNE. This was also a compelling motivator for participants.

I don't have as many hypos's, that's another one. I went around with a bottle of 7-Up in my bag constantly.(P42-35 6 weeks)

I mean I'm following the principles of DAFNE, and my HbA1c is down in the last 6 months from what it was, so I mean it must be working to some extent, but I mean I'm not having any problems or I had no hypos or I've nothing like that...(P44-42 12 month

Maintaining motivation over time therefore was important because it determined the extent to which participants persevered and utilised knowledge to self manage. Six months was an important point for participants as motivation was for some at its lowest.

### Continued Responsive Support

Participants also described the importance of having support, while they emphasised the need for family support and understanding, their accounts focused mainly on the support of other DAFNE participants and health professionals. This theme divides into two subcategories; group support and health professional support.

#### Group Support

Initially for many participants the support of other DAFNE participants was seen as really important. Sharing experiences during the courses really helped and they felt they learnt from the expertise of other participants. It was clear that group support was important and that there was a real sense of empathy and fun:

...*I found them brilliant...I found them really sociable, outgoing...it wasn't like an exhaustive activity or day, we did have some fun in the middle of it. It was very useful in that you met people who had the same experiences, and people who offered advice on certain topics... I suppose it was extra kind of ideas and experiences of other people that you could associate with... (P31-0042 6 weeks)*

There was real interest in how group members got on with DAFNE initially and participants were keen to find this out at the first post DAFNE meetings, which took place approximately 6 weeks after the DAFNE course. At times there were common issues and therefore group discussion of blood glucose levels were relevant but on the whole this component became less rather than more important as time went on. Half of the participants were invited to extra group sessions at approximately 6, 12 and 18^th ^months after the initial DAFNE course. Most of these participants reported that they rarely met outside the group and interest in the group appeared less important as time went on. Over time for many participants there was a shift from working and learning with others to solving my issues and the need to focus on me again.

*"Not really I mean at this stage I'm not sure how much more group work would actually be of benefit to me" (P9-096 12 months)*.

*"I think I find now after all this time the group session there's not as much said as before, because it's the same kind of people having the same kind of problems. And you kind of think now it might be better off just to speak to the expert rather than listen to - again like in the beginning it was - you learnt an awful lot from everybody else, but now I don't think so much now"(P13-100 12 months)*.

#### Health Professional Support

In contrast, support from health professionals was enduring over time, what participants valued most was responsive support that focused on collaborative decision making. Working DAFNE out in real life was described by some participants as problematic and they found that having a health professional who knew them and who understood DAFNE principles was crucial. Participants described how the ability to talk through issues and problems with a health professional really made a difference:

Knowing that there's back-up there, that I can ring if I get a problem, I think that helps me, rather than just thinking, there's no-one I can talk to... just, it's not working, forget it. I think... that helps motivate me...I just rang after the course to say that my sugars were going very well, so I rang to thank them (P13-95 6 weeks)

Some participants, therefore, linked support and motivation directly and suggested that having support really made a difference to sustaining DAFNE. Responsive support was particularly important at six months for this was the point at which some participants felt particularly de-motivated and frustrated. Participants suggested that access to educators was crucial because they sometimes needed help to solve problems that they were struggling with. What was important for them was that there was someone to talk the issues over with so that they could come to a decision.

..the support afterwards. In between clinics, it's just a case you do need something - or you're just not sure of something, or you just need somebody to say "yes go do it it's the right thing to do" (P13-001 6 months)

Participants suggested that DAFNE results in a shift from being told what to do, to deciding what to do and that responsive support for decision making was important to them. Some participants were able to access responsive support and suggested that this kept them on-track. However some participants found that support was not always consistently there, one participant who was struggling with DAFNE at six months had tried to get help but found it lacking, so he decided by the twelve month interview that a return to pre DAFNE management was his only option.

"I had a few major problems there come February and March. I was always getting a very high result in the morning, and I was able to communicate with the hospital's diabetic nurses there on two occasions, and they got me to do a 3.a.m blood test to see how things were going during the night, and then eventually all communication seemed to stop with emails, and there was no reply to my telephone calls. So eventually I just became proactive, and I came off the DAFNE system, and I went back on to my old system, which solved the problem for me. (P13-043 12 months)

It was evident at six months that the same participant was struggling with DAFNE

*They were extremely high (blood glucose levels) - like some were in the 20s for no reason I could explain...at the end of the day...I'd say I'm less motivated because I'm giving myself more injections a day than I was...and it's more or less for the same quantity of food that I'm eating (P13-042 6 months)*.

Six months was, for many participants, the time when motivation dipped most and therefore when support was most important. At twelve months support for decision making was still important but participants were more able to manage and accept this uncertainty.

I've worked most of the DAFNE things through at this stage, now you know, but everything seems to be you know - is stable. The only thing is ...like that getting' readings that there's no apparent reason for, you know...I know that what they're telling us has been medically proven and scientifically proven that basically you know that the one unit and all the rest of it will raise it 2 to 3 units, you know etc. But I think they need to be aware that there are times when, you know, there will - things will go wrong, and they aren't, you know, there's no explanation for it. (P 20-733 12 months)

And - ah - like it was 11 last night going' to bed so I corrected by one unit, and then when I was doing' that I thought well I'll have a chocolate - well I didn't have a chocolate digestive, but plain - you know the plain digestive, and I'd another unit for that, you know, and it worked, and as I say, you know, there's other times that it doesn't work, and I don't know what I do that it's any different sort of thing, you know. But thankfully my lows through the night, and things like that are few and far between. (P 020-773 12 months)

The need for support therefore remains over time, but responsive support is particularly important following the DAFNE programme and throughout the first six months.

### Study limitations

Longitudinal analysis is not a common qualitative method. It is therefore less structured with less guidance on how it should be done. It is not known if the approach taken is the best approach therefore a detailed description of what was done was given. With longitudinal research there is also a challenge maintaining a sample over time. In this study only two participants were lost to follow up. This occurred at the 12 month interview and was due to illness, it is not know if these participants' experiences differed from those who were interviewed.

## Discussion

This study focused on examining the factors that influenced persons with T1DM ability to assimilate and apply DAFNE principles over a 12 month period and how participants felt that these factors changed over time. Four factors were identified, embedded knowledge, enduring motivation, being empowered and continued responsive support.

It is generally agreed that knowledge is a prerequisite to empowerment and is essential to effective diabetes self management. While many researchers have found that SEP leads to improvements in knowledge [9,11,12] few have examined how this knowledge influences self management over time. This research reveals that following DAFNE, all participants felt they had more knowledge and, over time, many had begun to embed this knowledge into daily life giving them the power to choose what and when they wanted to eat. However such choices came at a price and some participants felt that although DAFNE offered freedom and a promise to 'eat what you want', there were consequences to this over time, in terms of potential weight gain and more frequent insulin injections. Nevertheless, embedded knowledge was key to effective self management in the long term as it enabled flexibility and contributed to an overall sense of empowerment. However, building knowledge takes time and confidence in using this knowledge requires practice and only grows with rehearsal. Health professionals therefore need to tell people that carbohydrate counting takes time to master and that embedding knowledge to effectively self manage their diabetes requires practice and may take up to a year to develop.

The way in which knowledge was delivered and participants were supported were also identified as factors which governed empowerment and self management over time. In this study participants reported that the initial DAFNE group sessions were really important and the knowledge and support gained from sharing experiences and learning from others was invaluable as it enhanced learning and empowerment. This finding is consistent to that found by Lawton & Rankin [37]. However, in this study few participants maintained contact with their group outside of the educational sessions. Most participants reported that the group education sessions became less important over time as participants required individual one to one responsive practical support and advice available as needed, focusing on their unique concerns. These findings are substantiated in other studies [8,48,49]. In particular, participants in this study reported that they wanted timely access to the right health professional when they were making real efforts to change but were being hampered by a transient problem they did not know how to manage. The need for timely support to resolve crises that threaten patients' ability to self manage has also been highlighted by other writers [33,36]. In addition similar to other studies, the six month time interval was critical [19]. Participants felt particularly de-motivated when, having adhered to DAFNE and having worked hard at trying to get it right, some still had unexplained high blood sugars at 6 months. The need to embed ongoing responsive support strategies as part of SEP, particularly at the six month period is therefore crucial if advances made by patients during the initial educational programme are to be maintained over time.

Preventing complications was an enduring motivator across time for all participants. Therefore their perception that they had experienced fewer hypoglycaemic episodes since commencing DAFNE was an important motivator. Some participants in this study reported that initially they thought that adhering to DAFNE would give them freedom to eat as they wished, while simultaneously guaranteeing lower blood glucose levels and thereby less complications. However at 6 months they realised that sometimes unexplained factors interfered with control. Some participants responded to this challenge more positively than others and adopted a trial and error approach with surprising tenacity. They tended to be problem solvers, readily taking on personal responsibility to self manage, and they appeared to relish the challenge, in the hope of 'getting it right' eventually. These participants sometimes exhibited characteristics similar to 'proactive managers' [50], and 'engaged and adjusting' patients [48]. At the 12 month follow up in this study these participants seemed to have accepted the fact that uncertainty was part of the self management process and that 'perfect' blood sugar levels were not always attainable and they seemed to accept this as reality for them, and continued with DAFNE. For others it was clear that over time DAFNE principles were being followed but with less experimentation in terms of changes in food, background insulin adjustment or meal times as they were uncomfortable with the trial and error approach. These participants often ate the same food and had the same mealtimes preferring routine and structure to their lives. Likewise Lawton et al [60] found that changes to dietary intake following DAFNE was relatively minor as patients soon realised that greater dietary freedom and choice brought new problems and restrictions. This UK qualitative longitudinal study explored whether patients changed the type of food they consumed following DAFNE.

A few participants perceived that the increased attention they gave to managing their diabetes over the past 12 months meant they were far more conscious of 'being a diabetic' and felt that diabetes now dominated their whole life and they found this disruptive and burdensome. From a health professional perspective this may not necessarily be a negative change in that one would hope that this focus would lead to better control. However, it is likely that these participants may in time become discouraged and these negative feeling may make them less likely to continue to adhere to DAFNE. Again there is a need for DAFNE educators to clearly explain and communicate to participants that familiarisation with DAFNE and applying these principles in the real world of everyday living takes time. Like learning any new skill it will require more focused attention and concentration at the outset but with time, and in some instances this may take over 12 months, this should become easier. Early identification of participants who are feeling overwhelmed by DAFNE is therefore required so that additional support and encouragement can be introduced in a timely, resource effective and responsive manner.

In this study it was found that the broader context and reality of life within which diabetes is managed impacted on people's ability to self management over time. Over the 12 month period, motivation was affected by various social demands; for example, caring for seriously ill relatives and other overwhelming life events and this reduced participants' ability and motivation to self manage their diabetes. The impact of life course events on people's ability to optimally self mange their diabetes over time was also identified by others ([8,61,36]. Having more resource to health professionals who really know the person and understand their unique circumstances may help support, motivate and empower them to adhere to good self management principles when faced with such day to day challenges

## Conclusions

The findings reveal that it takes time for behaviours to change, and become embedded into daily life. This study found that after the initial group education sessions, access to responsive support is really important, making the difference over time, to adhering to DAFNE self management principles. The support of knowledgeable health care professional may be, as suggested by Paterson et al. [62], a dynamic and lifelong need. However the intensity and extent of this need or support may diminish, further studies are needed to examine these issues. Finally, most studies focus on HbA1c as a marker of success yet hardly any demonstrate consistent glycaemic control over time. In addition few have examined participants' ability to assimilate SEP principles over time. This study helps to fill this void and in so doing identifies embedded knowledge, enduring motivation and continued responsive support, as vital factors influencing participant's ability to feel empowered and to self manage their diabetes over time.

## Competing interests

The authors declare that they have no competing interests.

## Authors' contributions

DC, KM, JL, FFW and SD developed the study design. DC and KM collected the data and drafted the article. DC, KM, JL, FFW conducted the data analysis. JL FFW & SD critically reviewed the paper for important intellectual content. All authors read and approved the final manuscript.

## Pre-publication history

The pre-publication history for this paper can be accessed here:

http://www.biomedcentral.com/1471-2458/11/672/prepub
